# Assessment of endothelium and inflammatory response at the onset of reperfusion injury in hand surgery

**DOI:** 10.1186/1476-9255-9-18

**Published:** 2012-05-14

**Authors:** Pranitha Kamat, Bettina Juon, Brigitte Jossen, Thusitha Gajanayake, Robert Rieben, Esther Vögelin

**Affiliations:** 1 Department of Clinical Research, University of Bern, Murtenstrasse 50 3008, Bern, Switzerland; 2 Clinic for Plastic- and Hand Surgery, Inselspital, Bern University Hospital, Bern, Switzerland

**Keywords:** Tourniquet, Hand surgery, Ischemia, Reperfusion injury, Cytokines, Complement, Endothelium, Glycocalyx

## Abstract

**Background:**

Activation of the endothelium, complement activation and generation of cytokines are known events during ischemia-reperfusion (I/R) that mediate tissue injury. Our aim was to elucidate their respective participation at the onset of the reperfusion phase. Tourniquet application in hand surgery causes short-term ischemia, followed by reperfusion and was therefore used as the model in this study.

**Methods:**

Ten patients were included in the study after obtaining informed consent. A tourniquet was placed on the upper arm and inflated to 250 mmHg for 116 ± 16 min, during which the surgery was performed. Venous blood and tissue samples from the surgical area were taken at baseline as well as 0, 2, and 10 min after reperfusion and analyzed for the following parameters: Endothelial integrity and/or activation were analyzed by measuring heparan sulfate and syndecan-1 in serum, and vWF, heparan sulfate proteoglycan as well as CD31on tissue. Complement activation was determined by C3a and C4d levels in plasma, levels of C1-inhibitor in serum, and IgG, IgM, C3b/c, and C4b/c deposition on tissue. Cytokines and growth factors IL-5, IL-6, IL-7, IL-8, IL-10, IL-17, G-CSF, GM-CSF, MCP-1, TNFα, VEGF, and PDGF bb were measured in the serum. Finally, CK-MM levels were determined in plasma as a measure for muscle necrosis.

**Results:**

Markers for endothelial activation and/or integrity as well as complement activation showed no significant changes until 10 min reperfusion. Among the measured cytokines, IL-6, IL-7, IL-17, TNFα, GM-CSF, VEGF, and PDGF bb were significantly increased at 10 min reperfusion with respect to baseline. CK-MM showed a rise from baseline at the onset of reperfusion (p < 0.001) and dropped again at 2 min (p < 0.01) reperfusion, suggesting ischemic muscle damage.

**Conclusions:**

In this clinical model of I/R injury no damage to the endothelium, antibody deposition or complement activation were observed during early reperfusion. However, an increase of pro-inflammatory cytokines and growth factors was shown, suggesting a contribution of these molecules in the early stages of I/R injury.

## Background

Ischemia / reperfusion (I/R) injury is a common source of pathology in many vascular diseases. Mechanisms underlying I/R injury have been studied extensively and are known to engage a spectrum of pathways. Elucidating the key molecules involved in triggering the entire process of injury is important to help develop targeted therapy to attenuate I/R injury in its early stages.

In any vascularized organ or tissue, a monolayer of endothelial cells (EC) forms the interface between blood and the surrounding tissue. Among other factors, the glycocalyx covering the endothelium, plays a critical role in maintaining the homeostasis of the blood vessel wall [1]. The conditions during I/R cause this glycocalyx layer to partly shed [2], which occurs already during ischemia and more significantly during reperfusion [3]. Glycocalyx shedding activates the endothelium by transforming it into a pro-inflammatory and pro-coagulant phenotype [4], thereby propagating injury. Moreover, the glycocalyx acts as an interface between blood and tissue, forms receptors for many inflammatory molecules including cytokines and therefore participates in inflammation [5,6]. Shedding of the glycocalyx after 2 min of reperfusion has been shown in humans [7], but the respective study was based on a setting of cardiopulmonary bypass and data on I/R induced shedding of the glycocalyx in smaller, peripheral blood vessels are lacking.

Complement activation leads directly to tissue necrosis and trafficking of immune cells. Various knockout animal models have illustrated the participation of natural antibodies and complement in propagation of I/R injury [8,9]. The importance of complement in I/R injury has been reviewed [10]. Complement thus has the potential to significantly contribute to early reperfusion injury.

The model used in this study was that of tourniquet-induced I/R injury. Tourniquet application in extremity surgery is a prerequisite to provide a blood-less environment during surgery. The blood flow in the ischemic limb is restored after surgery by releasing the tourniquet. Use of the tourniquet thereby comes with the risk of I/R injury. Clinically, this manifests as pain, swelling, prolonged hypoesthesia of peripheral nerves, tissue necrosis along with systemic effects, which the surgeons try to avoid by limiting tourniquet times to a maximum of 2 hours [11-15]. Several studies in humans have been dedicated to understanding I/R injury due to tourniquet application in upper and lower limbs. These studies have shown the involvement of radical oxygen species (ROS) [16], expression of adhesion molecules [17], recruitment of activated leukocytes [18] and thereby progression of inflammation. The process of I/R injury in skeletal muscle has been extensively studied [19] and reviewed [20]. However, the role of cytokines during early reperfusion is still unclear and yet to be investigated in upper limb I/R injury. This would be important, as cytokines apart from trafficking immune cells are known to cause shedding of the endothelial glycocalyx [21].

Based on the above-cited literature, including our own studies, we hypothesized that the endothelium together with the innate immune response including natural antibodies, complement, cytokines and growth factors, would be involved in the very early reperfusion phase. The aim of this study was to assess the involvement and relative contribution of these different factors in the initial phase of reperfusion injury.

## Methods

Ten patients undergoing elective hand surgery under tourniquet application were included in the study upon approval by the ethical committee of the Canton of Bern, Switzerland (reference number 033/09). Informed consent from the patients was obtained prior to the study. Exclusion criteria were trauma, anticoagulation, rheumatoid disease, age under sixteen years and diabetes. Patient details with their age, gender, main disease for operation, anatomical location of the operation, disorders, drugs given, tourniquet time, etc. are given in Table 1.

**Table 1:** Patient details

**No.**	**Age/ Gender**	**Main disease for operation**	**Location of operation**	**Cardiovascular disorder**	**Other disorders**	**Angiological disorder**	**Smoker**	**Drugs (/day)**	**Statins or Aspirin**	**Tourniquet time (min)**
1	35/ M	Tendon adhesions after complex reconstruction of left hand	Dorsal hand	No	Mild hepatitis	No	No	No	No	124
2	43/ F	Flexor tendon rupture left hand	Volar hand	No	No	No	No	No	No	112
3	33/ F	Tendon adhesions after complex reconstruction of right hand	Dorsal hand	No	No	No	No	No	No	118
4	29/ M	Ulnar nerve compression left elbow	Elbow	No	No	No	No	No	No	78
5	48/ M	Ulnar nerve compression left elbow	Elbow	No	No	No	Yes	No	No	105
6	60/ F	Rhizarthrosis right hand	Dorsal hand	No	No	No	Yes	Pantoprozole	No	128
7	47/ M	Posttraumatic arthritis of distal radio ulnar joint of left hand	Dorsal hand	Hypertension	Epilepsy	No	No	Lamotrigine, Amitriptyline Sodium valproate, Carvedilol, Chlorthalidone, Captopril / Hydrochlorothiazide	No	117
8	62/ F	Rhizarthrosis right hand	Dorsal hand	No	No	No	No	No	No	132
9	37/ M	Scaphoid non union right hand	Volar hand	No	No	No	No	No	No	127
10	68/ F	Rhizarthrosis right hand	Dorsal hand	Hypertension, 16 years prior PTCA and stent	Hypothyroidism	No	No	Atenolol, Ranitidine, Levothyroxine (0.1 mg), Estradiol (1 mg)	Aspirin	122

The tourniquet device was the A.T.S. 2000 automatic Tourniquet System (Zimmer, Inc. Warsaw, IL, USA) with a low profile cuff and accurate pressure monitoring. Before the cuff was applied on the upper arm a cuff sleeve to reduce shearing of soft tissue was used. After standardized disinfection with 0.5% chlorhexidin and sterile dressing surgery, samples were collected for the study.

### Sample collection

Venous blood leaving the surgical area was collected from the cubital vein, distal to the tourniquet, with a sterile syringe. Collected blood was immediately transferred into S-monovette tubes (Sarstedt AG, Nümbrecht, Germany) containing EDTA (1.6 mg/ml) to obtain EDTA-plasma, and to tubes containing a clotting activator (glass pearls) to obtain serum. Samples were collected before application of tourniquet (baseline), and immediately after release of the tourniquet (at 0, 2 and 10 min after reperfusion). Samples were kept on ice until centrifugation at 3000 rpm for 10 min. Serum and EDTA-plasma were stored in aliquots at −80°C until use.

Subcutaneous tissue samples containing blood vessels were taken from the surgical area distal to the tourniquet, and therefore within the ischemic area. Sampling was done immediately after the application of tourniquet (baseline), just before releasing the tourniquet (end ischemia) and 10 minutes after release of tourniquet (10 min reperfusion). Biopsies were fixed in 2% formaldehyde for 24 hours and then transferred into 18% sucrose for 15 hours. They were embedded in Shandon M1 embedding matrix (Thermo Scientific, Inc., Geneva, Switzerland) and stored at -20°C until sectioned.

### Markers for EC integrity/activation and detection of complement activation on tissue

Free float technique was used for immunostaining of tissue samples. In brief, 30 μm thick cryosections were cut from each sample and treated with TBS-Triton X100 for 15 min. EC integrity/activation were assessed by mouse anti-human heparan sulfate proteoglycan (HSPG; Abcam plc., Cambridge, UK), mouse anti-human von Willebrand factor (vWF; DAKO, Glostrup, Denmark), mouse anti-human CD31 (eBioscience, Inc., San Diego, CA, USA). As secondary antibodies we used Dylight 488 labeled donkey anti-mouse (Jackson ImmunoResearch Laboratories, Inc., West Grove, PA, USA), Cy3-labeled donkey anti-mouse (Jackson ImmunoResearch) and FITC labeled rabbit anti-mouse (DAKO).

The following antibodies were used to determine natural antibody binding and complement activation: Cy3 labeled goat anti-human IgG (KPL, Inc., Gaithersburg, MD, USA), allophycocyanin labeled goat anti-human IgM (Open Biosystems / Thermo Scientific), FITC labeled rabbit anti-human C3b/c (DAKO), and FITC-labeled rabbit anti-human C4b/c (DAKO).

### Scoring of stained tissue sections

The sections had highly heterogeneous vessel sizes approximately ranging between 12 – 100 μm. The variation due to vessel size and inter-batch differences during the staining procedure were overcome by the following scoring protocol: Sections of skeletal muscle from the hand of a healthy human were used as common control in every staining batch. Images of a representative blood vessel were taken under the confocal microscope from all sections including the common control. With Imaris software (Bitplane AG, Zurich, Switzerland), the area of the vessel was calculated from the image, and a histogram of the color channel of interest for the same area was obtained. The histogram of the common control was used to normalize histograms of other tissues from the same batch. This enabled sections from different batches to be comparable. Area under the curve (AUC) values were obtained from the normalized histogram for each tissue and divided by the area of the vessel to obtain a final score for the section. Sections were then compared with their final scores, which considered the size of the vessel and the intensity of staining within that vessel. Sections were blinded at all times for the analysis.

### Analysis of EC integrity/activation and complement activation in venous blood

ELISA kits were used to determine the concentrations of heparan sulfate (HS) and syndecan-1 as markers of EC activation. Kits for C3a, C4d and functional C1-inhibitor were used to analyze complement activation. Assays were performed according to manufacturers' protocols and concentrations were determined by comparing with standards provided with the kit. HS (Seikagaku Corp., Tokyo, Japan) and syndecan-1 (Diaclone, Gene-Probe Inc., San Diego, CA, USA) were measured in blood serum samples. C3a and C4d (Quidel Corp., San Diego, CA, USA) were measured in EDTA plasma samples and functional C1-inhibitor (Quidel Corp., San Diego, CA, USA) measured in blood serum.

### Analysis of cytokines in circulation

A multiplex immunoassay, consisting of fluorescent microspheres conjugated with a monoclonal antibody specific for a target protein, was used to detect an array of cytokines. Kits for IL-5, IL-6, IL-7, IL-8, IL-10, IL-17, G-CSF, GM-CSF, MCP-1, TNFα, VEGF, and PDGF bb were purchased from Bio-Rad (Bio-Rad Laboratories, Inc., Hercules, CA, USA) and multiplex analysis was performed on a Bio-Plex 100 system (Bio-Rad). Assays were performed according to manufacturer’s instructions. Briefly, plasma was diluted 1:2 and incubated with antibody-coupled beads. Complexes were washed and then incubated with biotinylated detection antibody followed by streptavidin-phycoerythrin prior to assessing titers of cytokine concentration. Recombinant cytokines were used to establish standard curves. Analyte concentrations were calculated using the Bio-Plex Manager 4.0 Software (Bio-Rad).

### Measurement of skeletal muscle injury

An ELISA kit for measuring skeletal muscle creatine kinase (CK-MM, from USCN Life Sciences, Wuhan, China) was used to measure skeletal muscle injury. The assay was performed according to the manufacturers' protocol and samples were analyzed in duplicates for each time point. Concentrations were determined by comparing with standards provided with the kit.

### Statistical analysis

All data was initially checked for normality by Kolmogorov-Smirnov test. Time dependant changes were tested by Repeated Measures ANOVA test with confidence interval set to 95%. Bonferroni's multiple comparison test was used as a post test. When only two time points had to be statistically compared, two tailed paired t-test was used with the level of significance set to 95%. All statistics calculations were done in Prism 4.0a for Macintosh (GraphPad Software, Inc., La Jolla, CA, USA).

## Results

The average tourniquet time for the 10 patients was 116 ± 16 minutes.

### Shedding of glycocalyx and EC integrity/activation

**Figure 1:**
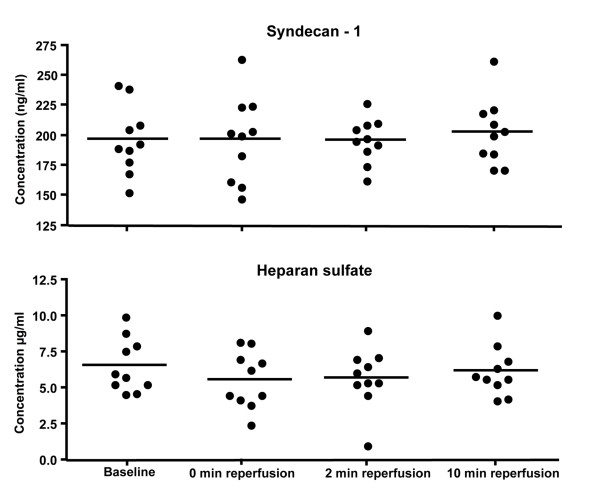
**Serum levels of Heparan sulfate and Syndecan-1 .** Serum levels of HS and syndecan-1 were measured by ELISA. Samples from baseline were compared with 0, 2, and 10 min reperfusion. No significant changes between time points were found by Repeated Measures ANOVA, n = 10.

Heparan sulfate and syndecan-1 were measured by ELISA. Figure 1 shows each patient as an individual dot and the mean values are represented with a dash. Given below in brackets are the mean and standard deviation values for the ten patients at every time point. Statistical analysis showed no time dependent change in levels of HS at baseline (6.5 ± 1.9 μg/ml), 0 min reperfusion (5.6 ± 1.9 μg/ml), 2 min reperfusion (5.5 ± 2.1 μg/ml) and 10 min reperfusion (6.2 ± 1.8 μg/ml). Similarly, syndecan-1 levels showed no time dependent changes from baseline (196.4 ± 28.4 ng/ml) to 0 min reperfusion (196.5 ± 35.8 ng/ml), 2 min reperfusion (196.0 ± 18.6 ng/ml), and 10 min reperfusion (102.7 ± 27.5 ng/ml).

**Figure 2:**
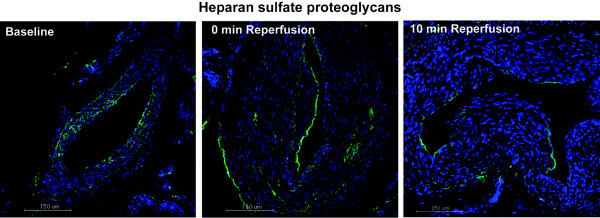
**Immunofluorescence staining for HSPG.** Tissue samples from baseline, end ischemia and 10 min reperfusion were analyzed by Immunofluorescence for HSPG. Pictures are representative for the shown time points. No statistical differences in HSPG expression were found by Repeated Measures ANOVA, n = 10. Green = HSPG, blue = DAPI staining of nuclei. Scale bars = 150 μm.

The endothelial lining of blood vessels was studied by immunostaining the tissue sections for HSPG, vWF and CD31 as markers of EC integrity/activation. Table 2 shows mean values ± standard deviations for each marker at the different time points. Statistics showed no significant differences in staining intensity, confirming no loss of endothelial integrity or activation of the EC until 10 min reperfusion. Representative pictures for HSPG at the different time points are shown in Figure 2.

**Table 2:** Immunofluorescence staining for markers of EC integrity / activation

Markers of EC integrity / activation	Baseline	End ischemia	10 min reperfusion	P value
HSPG	0.45 ± 0.16*	0.44 ± 0.14	0.58 ± 0.11	n.s.
vWF	0.34 ± 0.37	0.64 ± 0.64	0.60 ± 0.49	n.s.
CD31	2.20 ± 3.99	0.61 ± 0.83	0.51 ± 0.57	n.s.

*Values are mean ± standard deviation of immunofluorescence scores (quantitation by Imaris software), n = 10.

### Binding of antibodies and activation of complement system

**Figure 3:**
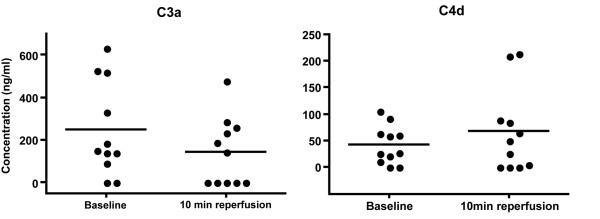
**Plasma levels of C3a and C4d.** Plasma levels of the complement activation markers C3a and C4d were measured by ELISA. Values at baseline and after 10 min reperfusion were not statistically different as shown by Paired t-Test, n = 10.

**Figure 4:**
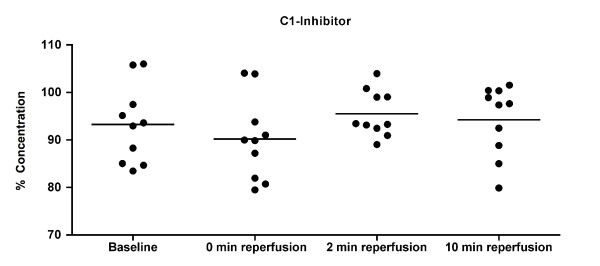
**C1-Inhibitor in serum.** Functional C1-inhibitor levels in serum were measured by ELISA. Values were not significantly different between time points as shown by Repeated Measures ANOVA.

Products of complement activation were measured by ELISA at baseline and after 10 min of reperfusion in EDTA plasma samples. Figure 3 shows each patient as an individual dot and the mean represented with a dash. Given below in brackets are the mean and standard deviation value of the ten patients at every time point. The paired t-test showed no significant differences between the levels of C3a at baseline (247.0 ± 221.5 ng/ml) and at 10 min reperfusion (144.7 ± 161.1 ng/ml) and of C4d at baseline (42.5 ± 35.9 ng/ml) and 10 min reperfusion (67.6 ± 78.4 ng/ml). The levels of functional C1-inhibitor were expressed as percentage of normal levels in human blood and measured at baseline (93.2 ± 8.2%), 0 min reperfusion (90.2 ± 8.7%), 2 min reperfusion (95.5 ± 4.8), and 10 min reperfusion (94.2 ± 7.4). In line with the C3a and C4d results, also no significant differences were found for the levels of C1-inhibitor (Figure 4).

**Figure 5:**
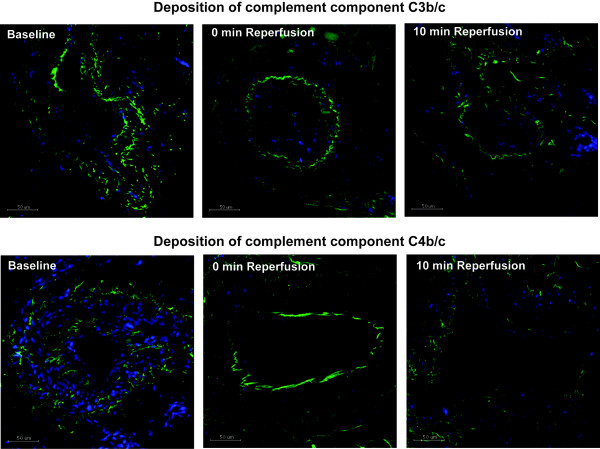
**Immunofluorescence staining for C3b/c and C4b/c.** Tissue samples from baseline, end ischemia and 10 min reperfusion were analyzed by Immunofluorescence for deposition of the complement components C3b/c and C4b/c. Pictures are representative for the time points and no significant differences were found by Repeated Measures ANOVA, n = 10. Green = C3b/c / C4b/c, blue = DAPI staining of nuclei. Scale bars = 50 μm.

In the tissue samples, deposition of IgM, IgG, C3b/c and C4b/c were analyzed by immunostaining. Table 3 shows mean values ± standard deviations for each antigen at the different time points. Statistical analysis revealed no significant differences for antibody- or complement deposition in tissue between baseline and 10 min reperfusion. Pictures representative for C3b/c and C4b/c deposition are shown in Figure 5.

**Table 3:** Immunofluorescence staining for markers of complement activation

Antibody and complement deposition in tissue	Baseline	End ischemia	10 min reperfusion	P value
IgG	1.03 ± 9.60*	0.81 ± 0.60	0.50 ± 0.40	n.s.
IgM	0.02 ± 0.02	0.06 ± 0.08	0.03 ± 0.03	n.s.
C3b/c	0.12 ± 0.13	0.12 ± 0.22	0.13 ± 0.22	n.s.
C4b/c	0.16 ± 0.10	0.29 ± 0.46	0.22 ± 0.31	n.s.

*Values are mean ± standard deviation of immunofluorescence scores (quantitation by Imaris software), n = 10.

### Cytokine levels

**Figure 6:**
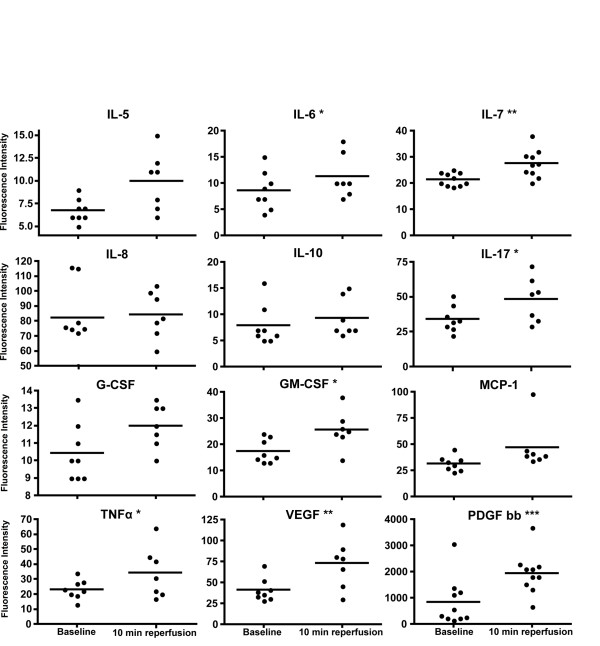
**Measurement of cytokines and growth factors in serum.** The cytokines IL-5, IL-6, IL-7, IL-8, IL-10, IL-17, G-CSF, GM-CSF MCP-1, TNFα, VEGF and PDGF bb were measured by Bio-Plex assay. Baseline samples were compared with 10 min reperfusion samples for each cytokine. *p < 0.05, **p < 0.005, ***p < 0.0005 (two-tailed Student's t-test), n = 10.

An array of cytokines and growth factors, namely IL-5, IL-6, IL-7, IL-8, IL10, IL17, G-CSF, GM-CSF MCP-1, TNFα, VEGF and PDGF bb, were measured by Bio-Plex assay. Because several of the measurements revealed low values outside of the range of the standard curves, raw fluorescence intensity values were used for statistical analysis, rather than the calculated concentrations of the respective cytokines. Figure 6 shows each patient as an individual dot with the mean represented by a dash. The 10 min reperfusion samples were significantly higher than baseline for IL-6 (p = 0.024), IL-7 (p = 0.006), IL-17 (p = 0.034), GM-CSF (p = 0.017), TNFα (p = 0.046), VEGF (p = 0.007), and PDGF bb (p = 0.0001) as analyzed by paired t-test.

### Skeletal muscle injury

**Figure 7:**
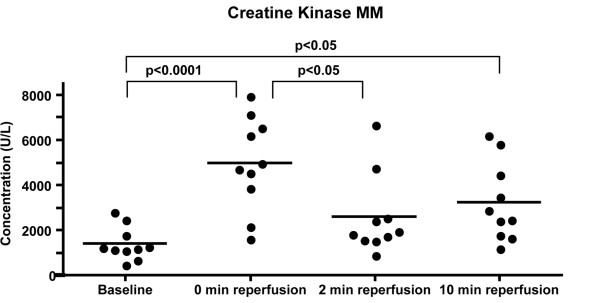
**Plasma levels of creatine kinase MM.** CK-MM was measured by ELISA and found to be significantly higher at the end of ischemia (0 min reperfusion) as compared to baseline. Levels dropped again during the reperfusion phase, but were still higher than baseline after 10 min reperfusion (as shown by Bonferroni’s post test for Repeated Measures ANOVA), n = 10.

Creatine kinase-MM was measured in plasma samples as a marker for skeletal muscle injury. Figure 7 shows each patient as an individual dot and the mean represented by a dash at every time point. The values given below are mean ± standard deviation. CK-MM values increased significantly from 2066 ± 1122 U/L at baseline to 5908 ± 1843 U/L at 0 min reperfusion (p < 0.001). At 2 min reperfusion the levels significantly dropped from 0 min reperfusion to 3504 ± 1855 U/L (p < 0.001). However, at 10 min reperfusion, CK-MM levels (4296 ± 1894 U/L) were still higher than at baseline (p < 0.01).

## Discussion

As an interface between the circulating blood and tissue it is not difficult to consider the EC to react immediately to pathophysiological processes during I/R. An important reaction of the endothelium is the shedding of its native, anticoagulant and anti-inflammatory surface layer, the glycocalyx [22]. The latter is composed mainly of heparan sulfate proteoglycans, among which syndecans as core proteins [7]. We therefore measured the levels of shed heparan sulfate, syndecan-1 in serum and expression of HSPG on tissue, which showed no changes from baseline to 10 min reperfusion. Together, these data support the notion that no significant activation of EC occurred in our setting of tourniquet-induced ischemia and reperfusion. This finding is in contrast to earlier reports, for example on patients undergoing coronary artery bypass grafting under extracorporeal circulation, where increased serum levels of syndecan-1 and HS were found as early as after 2 min of reperfusion [7]. In the latter study, however, the tubings and filters in the extracorporeal circulation circuit, which are known to lead to complement activation, may account for at least part of the observed EC activation.

Other markers of EC activation documented previously are CD31 and vWF [23,24], and we included detection of these antigens on tissue biopsies by immunostaining. Earlier studies by Huhges et al. [25,26], showed no significant increase in vWF plasma levels after 10 min and 30 min of ischemia and 15 min and 30 min reperfusion, respectively. Our study confirms this finding with no significant changes in vWF and CD31 expression in biopsies, which further indicate minimal EC activation or damage during early reperfusion. However, the study does not consider differences in the constitutive expression patterns of these antigens, which could vary due to heterogeneity in vessel size as shown for vWF [27].

Previous studies on murine skeletal muscle I/R injury models have identified natural IgM antibodies as a major initiator of the activation of the complement system, which was also shown to be causally related to the observed tissue injury [8]. We therefore analyzed deposition of IgM as well as IgG in the tissue. However, no significant differences between ischemia and reperfusion samples were found for these antibody isotypes. In line with the absence of antibody deposition, markers for complement activation C3b/c, C4b/c on tissue and C3a, C4d in plasma did not change until 10 min reperfusion. This suggests that no significant activation of the complement system occurred in our setting of tourniquet-induced ischemia and reperfusion injury. Also the levels of functional C1-inhibitor, which is the only known physiologic inhibitor of classical complement pathway activation [28], showed no changes from baseline to 10 min reperfusion.

Pro-inflammatory cytokines are secreted to preserve immune integrity and stimulate repair mechanisms to counteract the ongoing tissue damage [29,30]. The pro-inflammatory cytokines IL-1 and TNFα in particular are released by monocytes and macrophages during the acute phase of I/R injury and in turn stimulate the production of IL-6 [31]. In our study we found that TNFα and IL-6 were significantly elevated in serum at 10 min reperfusion as compared to baseline. This result is in line with two reports of tourniquet application in lower limb surgery, where a rise in IL-6 was shown at 2 h and 4 h reperfusion, respectively, in the draining blood of the ischemic limbs [19,32]. We also found a significant rise in the levels of IL-17 from baseline, highlighting the involvement of another pro-inflammatory molecule, which is known to induce EC to secrete cytokines.

Among the cytokines secreted by EC, IL-7 showed a significant rise in the 10 minutes reperfusion sample, but this was not the case for IL-8. Predominantly anti-inflammatory cytokines like IL-10 and IL-5 did not show any significant changes at 10 min reperfusion.

To our knowledge, the role of MCP-1 has not yet been studied in human skeletal muscle I/R injury. Studies on MCP-1 in other models have shown its involvement in both inflammation [33] and muscle regeneration [34]. Our data show no significant changes in the levels of MCP-1 until 10 min of reperfusion. On the other hand, an increased level of GM-CSF, but not of G-CSF, was found, probably due to the recruitment of monocytes at the site of ischemia.

VEGF [35] and PDGF bb [36] are growth factors that help in angiogenesis and formation of new blood vessels and are induced as a response to ischemia. In the present study we could also show this type of vascular responses to ischemia by significantly higher plasma levels of VEGF and PDGF bb at 10 min reperfusion.

Finally, CK-MM was measured as a marker for skeletal muscle injury [37]. The observed significant rise of the enzyme levels at end of ischemia (0 min reperfusion) as well as at 10 min reperfusion when compared to baseline indicates that a certain level of skeletal muscle injury is occurring in our model, despite the apparent absence of activation of complement and EC.

### Clinical significance of the study

As mentioned previously the use of tourniquet on a limb renders it to the risk of I/R injury which clinically manifests as pain, swelling, prolonged hypoesthesia of peripheral nerves, tissue necrosis along with systemic effects. For the formation of edema, which may lead to the feared compartment syndrome, EC activation and damage are key events. Their absence during the very early reperfusion phase (as shown in our study) may offer a window of opportunity to protect the endothelium and thus to prevent further reperfusion injury, for example by local use of endothelial cell protective substances which proved to be effective to prevent I/R injury in experimental myocardial infarction [38,39].

### Conclusions and limitations

The aim of this study was to assess the endothelium and the inflammatory response during the early phase of reperfusion. In this clinical model of tourniquet induced I/R injury, we show an active involvement of the inflammatory cascade with a rise in cytokines, mainly TNFα, IL-6, IL-17, IL-7, GM-CSF, PDGF bb and VEGF, during early reperfusion. Additionally a rise in CK-MM was observed indicating a certain degree of skeletal muscle injury post surgery. However, in contrast to our original hypothesis, no significant activation of EC or the complement system could be observed until 10 min reperfusion.

A limitation of the study is that we cannot account for the source of the increased cytokine production as observed, which could be local or systemic. Additional analysis of the same parameters in samples taken from the systemic circulation would have been helpful. The short follow-up of only 10 min during the reperfusion phase is a limitation of the study. Clinically, edema becomes apparent only several hours after surgery and additional studies with analyses of plasma samples after reperfusion times of 6 h or more need to be done. Another limitation of the study is the ischemia period with a rather wide standard deviation and the varying clinical background and underlying diseases of the patients. However, there is no scope for standardization of these two parameters in a clinical setting like the current study, and more uniform experiments in this direction will have to be conducted in animal models.

## Competing interests

The authors declare that they have no completing interests.

## Authors’ contributions

Designed the experiments / the study: PK, BJU, RR, EV. Analyzed and interpreted the data: PK, BJ, TG, RR. Collected data / did experiments for the study: PK, BJ. Enrolled patients/conducted the surgery: BJU, EV. Contributed to the writing of the paper: PK, BJU, TG, RR, EV. Agree with manuscript’s results and conclusions. All authors read and approved the final manuscript.

## References

[B1] Van Teeffelen JW Brands J Stroes ES Vink H Endothelial glycocalyx: sweet shield of blood vessels Trends Cardiovasc Med 2007 17 101 105 10.1016/j.tcm.2007.02.002 17418372

[B2] Mulivor AW Lipowsky HH Inflammation- and ischemia-induced shedding of venular glycocalyx Am J Physiol Heart Circ Physiol 2004 286 H1672 H1680 10.1152/ajpheart.00832.2003 14704229

[B3] Ward BJ Donnelly JL Hypoxia induced disruption of the cardiac endothelial glycocalyx: implications for capillary permeability Cardiovasc Res 1993 27 384 389 10.1093/cvr/27.3.384 7683973

[B4] Shibata S Sasaki T Harpel P Fillit H Autoantibodies to vascular heparan sulfate proteoglycan in systemic lupus erythematosus react with endothelial cells and inhibit the formation of thrombin-antithrombin III complexes Clin Immunol Immunopathol 1994 70 114 123 10.1006/clin.1994.1018 8299226

[B5] Wang L Fuster M Sriramarao P Esko JD Endothelial heparan sulfate deficiency impairs L-selectin- and chemokine-mediated neutrophil trafficking during inflammatory responses Nat Immunol 2005 6 902 910 16056228 10.1038/ni1233

[B6] Tanaka Y Kimata K Adams DH Eto S Modulation of cytokine function by heparan sulfate proteoglycans: sophisticated models for the regulation of cellular responses to cytokines Proc Assoc Am Physicians 1998 110 118 125 9542767

[B7] Rehm M Bruegger D Christ F Conzen P Thiel M Jacob M Chappell D Stoeckelhuber M Welsch U Reichart B Shedding of the endothelial glycocalyx in patients undergoing major vascular surgery with global and regional ischemia Circulation 2007 116 1896 1906 10.1161/CIRCULATIONAHA.106.684852 17923576

[B8] Austen WG Zhang M Chan R Friend D Hechtman HB Carroll MC Moore FD Murine hindlimb reperfusion injury can be initiated by a self-reactive monoclonal IgM Surgery 2004 136 401 406 10.1016/j.surg.2004.05.016 15300207

[B9] Zhang M Alicot EM Chiu I Li J Verna N Vorup-Jensen T Kessler B Shimaoka M Chan R Friend D Identification of the target self-antigens in reperfusion injury J Exp Med 2006 203 141 152 10.1084/jem.20050390 16390934 PMC2118091

[B10] Banz Y Rieben R Role of complement and perspectives for intervention in ischemia-reperfusion damage Ann Med 2012 44 1 13 10.3109/07853890.2010.532150 21254897

[B11] Mohler LR Pedowitz RA Lopez MA Gershuni DH Effects of tourniquet compression on neuromuscular function Clin Orthop Relat Res 1999 359 213 220 10078146 10.1097/00003086-199902000-00024

[B12] Lin LN Wang LR Wang WT Jin LL Zhao XY Zheng LP Jin LD Jiang LM Xiong XQ Ischemic preconditioning attenuates pulmonary dysfunction after unilateral thigh tourniquet-induced ischemia-reperfusion Anesth Analg 2010 111 539 543 10.1213/ANE.0b013e3181e368d2 20610550

[B13] Smith TO Hing CB The efficacy of the tourniquet in foot and ankle surgery? A systematic review and meta-analysis Foot Ankle Surg 2010 16 3 8 10.1016/j.fas.2009.03.006 20152747

[B14] Girardis M Milesi S Donato S Raffaelli M Spasiano A Antonutto G Pasqualucci A Pasetto A The hemodynamic and metabolic effects of tourniquet application during knee surgery Anesth Analg 2000 91 727 731 10.1213/00000539-200009000-00043 10960408

[B15] Sapega AA Heppenstall RB Chance B Park YS Sokolow D Optimizing tourniquet application and release times in extremity surgery. A biochemical and ultrastructural study J Bone Joint Surg Am 1985 67 303 314 3968122

[B16] Lindsay T Romaschin A Walker PM Free radical mediated damage in skeletal muscle Microcirc Endothelium Lymphatics 1989 5 157 170 2700374

[B17] Germann G Drucke D Steinau HU Adhesion receptors and cytokine profiles in controlled tourniquet ischaemia in the upper extremity J Hand Surg Br 1997 22 778 782 10.1016/S0266-7681(97)80447-0 9457587

[B18] Sutter PM Spagnoli GC Marx A Gurke L Troeger H Fricker R Harder F Heberer M Increased surface expression of CD18 and CD11b in leukocytes after tourniquet ischemia during elective hand surgery World J Surg 1997 21 179 184 discussion 185 10.1007/s002689900212 8995075

[B19] Huda R Solanki DR Mathru M Inflammatory and redox responses to ischaemia/reperfusion in human skeletal muscle Clin Sci (Lond) 2004 107 497 503 10.1042/CS20040179 15283698

[B20] Gute DC Ishida T Yarimizu K Korthuis RJ Inflammatory responses to ischemia and reperfusion in skeletal muscle Mol Cell Biochem 1998 179 169 187 10.1023/A:1006832207864 9543359

[B21] Chappell D Hofmann-Kiefer K Jacob M Rehm M Briegel J Welsch U Conzen P Becker BF TNF-alpha induced shedding of the endothelial glycocalyx is prevented by hydrocortisone and antithrombin Basic Res Cardiol 2009 104 78 89 10.1007/s00395-008-0749-5 18836678

[B22] Morgan MR Humphries MJ Bass MD Synergistic control of cell adhesion by integrins and syndecans Nat Rev Mol Cell Biol 2007 8 957 969 10.1038/nrm2289 17971838 PMC3329926

[B23] Woodfin A Voisin MB Imhof BA Dejana E Engelhardt B Nourshargh S Endothelial cell activation leads to neutrophil transmigration as supported by the sequential roles of ICAM-2, JAM-A, and PECAM-1 Blood 2009 113 6246 6257 10.1182/blood-2008-11-188375 19211506 PMC2699241

[B24] Datta YH Ewenstein BM Regulated secretion in endothelial cells: biology and clinical implications Thromb Haemost 2001 86 1148 1155 11816699

[B25] Hughes SF Hendricks BD Edwards DR Bastawrous SS Roberts GE Middleton J Hughes SF Hendricks BD Edwards DR Bastawrous SS Roberts GE Middleton JF Mild episodes of tourniquet-induced forearm ischaemia-reperfusion injury results in leukocyte activation and changes in inflammatory and coagulation markers J Inflamm (Lond) 2007 4 12 10.1186/1476-9255-4-12 17537260 PMC1890284

[B26] Hughes SF Hendricks BD Edwards DR Middleton JF Tourniquet-applied upper limb orthopaedic surgery results in increased inflammation and changes to leukocyte, coagulation and endothelial markers PLoS One 2010 5 e11846 10.1371/journal.pone.0011846 20676375 PMC2911384

[B27] Liu J Yuan L Molema G Regan E Janes L Beeler D Spokes KC Okada Y Minami T Oettgen P Aird WC Vascular bed-specific regulation of the von Willebrand factor promoter in the heart and skeletal muscle Blood 2011 117 342 351 10.1182/blood-2010-06-287987 20980682 PMC3037755

[B28] Storini C Rossi E Marrella V Distaso M Veerhuis R Vergani C Bergamaschini L De Simoni MG C1-inhibitor protects against brain ischemia-reperfusion injury via inhibition of cell recruitment and inflammation Neurobiol Dis 2005 19 10 17 10.1016/j.nbd.2004.11.001 15837556

[B29] Ferencik M Molecular and cellular mechanisms in inflammatory reactions Bratisl Lek Listy 1995 96 509 519 8620319

[B30] Ferencik M Stvrtinova V Endogenous control and modulation of inflammation Folia Biol (Praha) 1996 42 47 55 8831026

[B31] Akira S Isshiki H Nakajima T Kinoshita S Nishio Y Natsuka S Kishimoto T Regulation of expression of the interleukin 6 gene: structure and function of the transcription factor NF-IL6 CIBA Found Symp 1992 167 47 62 discussion 62–47 1385054 10.1002/9780470514269.ch4

[B32] Clementsen T Reikeras O Cytokine patterns after tourniquet-induced skeletal muscle ischaemia reperfusion in total knee replacement Scand J Clin Lab Invest 2008 68 154 159 10.1080/00365510701528587 17963155

[B33] Lu B Rutledge BJ Gu L Fiorillo J Lukacs NW Kunkel SL North R Gerard C Rollins BJ Abnormalities in monocyte recruitment and cytokine expression in monocyte chemoattractant protein 1-deficient mice J Exp Med 1998 187 601 608 10.1084/jem.187.4.601 9463410 PMC2212142

[B34] Shireman PK Contreras-Shannon V Ochoa O Karia BP Michalek JE McManus LM MCP-1 deficiency causes altered inflammation with impaired skeletal muscle regeneration J Leukoc Biol 2007 81 775 785 17135576 10.1189/jlb.0506356

[B35] Cherwek DH Hopkins MB Thompson MJ Annex BH Taylor DA Fiber type-specific differential expression of angiogenic factors in response to chronic hindlimb ischemia Am J Physiol Heart Circ Physiol 2000 279 H932 H938 10993752 10.1152/ajpheart.2000.279.3.H932

[B36] Iihara K Sasahara M Hashimoto N Uemura Y Kikuchi H Hazama F Ischemia induces the expression of the platelet-derived growth factor-B chain in neurons and brain macrophages in vivo J Cereb Blood Flow Metab 1994 14 818 824 10.1038/jcbfm.1994.102 8063877

[B37] Wu AH Perryman MB Clinical applications of muscle enzymes and proteins Curr Opin Rheumatol 1992 4 815 820 1457275

[B38] Banz Y Hess OM Robson SC Mettler D Meier P Haeberli A Csizmadia E Korchagina EY Bovin NV Rieben R Locally targeted cytoprotection with dextran sulfate attenuates experimental porcine myocardial ischaemia/reperfusion injury Eur Heart J 2005 26 2334 2343 10.1093/eurheartj/ehi421 16055495

[B39] Banz Y Hess OM Robson SC Csizmadia E Mettler D Meier P Haeberli A Shaw S Smith RA Rieben R Attenuation of myocardial reperfusion injury in pigs by Mirococept, a membrane-targeted complement inhibitor derived from human CR1 Cardiovasc Res 2007 76 482 493 10.1016/j.cardiores.2007.07.016 17825275

